# MicroRNA Regulation in Kidney Interstitial Fibrosis

**DOI:** 10.3390/epigenomes10010021

**Published:** 2026-03-16

**Authors:** Hirofumi Sakuma, Satoshi Kawaguchi, Yuya Kobayashi, Akiko Koizumi, Naoki Nakagawa

**Affiliations:** 1 Division of Cardiology and Nephrology, Department of Internal Medicine, Asahikawa Medical University, Asahikawa 078-8510, Japan; hsakuma210@asahikawa-med.ac.jp (H.S.); kyokui090073@gmail.com (Y.K.); koizumiakiko@asahikawa-med.ac.jp (A.K.); naka-nao@asahikawa-med.ac.jp (N.N.); 2 Department of Emergency Medicine, Asahikawa Medical University, Asahikawa 078-8510, Japan

**Keywords:** MicroRNA, kidney interstitial fibrosis, chronic kidney disease

## Abstract

MicroRNAs (miRNAs) are small non-coding RNAs that play central roles in post-transcriptional gene regulation and cellular homeostasis maintenance. Dysregulation of miRNA expression is increasingly recognized as a key contributor to tissue injury during the acute phase and to disease progression in the chronic phase. Chronic kidney disease (CKD) commonly progresses and ultimately leads to kidney failure through interstitial fibrosis, which is the final common pathway of CKD progression. Interstitial fibrosis is driven not only by fibroblast activation but also by phenotypic transitions in injured tubular epithelial cells, infiltrating macrophages, and peritubular capillary cells. These multifaceted cellular pathways induce and exacerbate interstitial fibrosis, and several miRNAs have been identified as important regulators of these pathways. In addition to fibrotic pathophysiological features, disease-specific dysregulation of miRNAs has been increasingly detected in various causes of CKD, including diabetic kidney disease, chronic glomerulonephritis, and nephrosclerosis. In this review, we provide an integrated overview of miRNA-mediated regulation in CKD, with particular emphasis on cell lineage functions within fibrotic pathways and disease-specific roles. Finally, we discuss the emerging potential of miRNAs as biomarkers and therapeutic targets for CKD and highlight future research directions.

## 1. Introduction

Non-coding RNAs (ncRNAs) are RNA molecules transcribed from DNA that are not translated into proteins. They contribute to several biological processes, including protein synthesis and intracellular transport, RNA maturation, DNA replication, and, most prominently, gene expression regulation. They are commonly classified per their length into small ncRNAs (<200 nucleotides) and long ncRNAs (>200 nucleotides). Among the small ncRNAs, microRNAs (miRNAs) are 18–22 nucleotides in length and are single-stranded transcripts that mediate gene silencing through RNA interference. Long ncRNAs include linear lncRNAs and circRNAs [1].

CKD affects approximately 10% of the global population [2]. Importantly, delaying early-stage CKD progression provides economic benefits and prevents the development of end-stage kidney disease (ESKD) and cardiovascular complications [3]. Although CKD arises from many heterogeneous diseases that irreversibly alter the function and structure of the kidney, such as diabetes, hypertension, and nephritis, it is characterized by progressive and irreversible nephron loss, microvascular damage, decreased regenerative capacity, inflammation, oxidative stress, and metabolic changes, ultimately leading to kidney failure [4,5,6]. In CKD progression, tubulointerstitial fibrosis is the key mechanism of kidney dysfunction, marked by excessive ECM deposition and loss of epithelial cells. This process is largely driven by activated myofibroblasts, which primarily derive from interstitial fibroblasts, with contributions from pericytes, circulating fibrocytes, and transitional processes involving tubular and endothelial cells [5,6,7,8]. However, the therapeutic options for kidney fibrosis are limited due to a lack of understanding of the underlying mechanism.

Recently, miRNAs have been focused on as new targets for various diseases. They interact with other types of ncRNAs, such as long ncRNAs and circRNAs, which are pivotal in cellular processes and disease development, including fibrosis. Now, most mammalian mRNAs are conserved targets of miRNAs, and their regulation in fibrosis development is significant. Therefore, elucidation of miRNA regulation of the fibrotic pathway, as key regulators, will fill a knowledge gap in the fibrotic mechanism and revolutionize the development of new, specific therapies for kidney fibrosis.

In this review, we summarized recent advances in the roles of dysregulated miRNAs, focusing on intercellular communication, mesenchymal transition, and fibroblast activation. We also highlighted potential miRNAs as novel biomarkers and antifibrotic therapeutic targets in various kidney diseases.

## 2. Methodology Literature Search

Here, we performed a literature search spanning over the past decade using electronic databases (PubMed and Google Scholar). We also screened relevant review articles to identify additional studies related to microRNAs and kidney interstitial fibrosis. Only articles published in English were included. The following keywords and their combinations were used: “kidney fibrosis,” “kidney interstitial fibrosis,” “microRNA” OR “miRNA,” “long non-coding RNA” OR “lncRNA,” “circular RNA” OR “circRNA,” “fibroblast,” “pericyte,” “myofibroblast,” “epithelial–mesenchymal transition (EMT),” “endothelial–mesenchymal transition (EndMT),” “macrophage-to-mesenchymal transition (MMT),” “diabetic kidney disease,” “IgA nephropathy,” “chronic kidney disease,” and “hypertensive nephropathy.”

## 3. Noncoding RNAs and the Kidney

NcRNAs are classified into two groups based on nucleotide length: small ncRNAs (sncRNAs) and lncRNAs. Most sncRNAs comprise miRNAs with an average of 22 nucleotides, whereas lncRNAs have over 200 nucleotides and include linear lncRNAs and circRNAs.

### 3.1. MiRNAs

MiRNAs regulate gene expression by binding to partially complementary sites, typically within the 3′ untranslated regions (3′UTRs) of the target mRNAs [9,10,11]. This interaction represses translation and promotes mRNA decay via an RNA-induced silencing complex. A single miRNA can target multiple mRNAs, while individual mRNAs can be regulated by several miRNAs [12,13]. Through these mechanisms, miRNAs influence several biological processes, including development, differentiation, cell proliferation, apoptosis, cancer metastasis, inflammation, and fibrosis [14]. MiRNAs were first described in 1993, and since then, over 2000 human miRNAs have been identified in miRBase [15]. Recent kidney research has identified various dysregulated miRNAs in acute kidney injury (AKI) and chronic kidney disease (CKD) [16,17,18,19,20,21,22]. Although there are currently no effective treatments that reverse established kidney interstitial fibrosis, miRNAs have emerged as promising mechanistic regulators and potential therapeutic targets [18,23]. Over the past decade, studies on miRNAs in kidney interstitial fibrosis have expanded rapidly, and numerous miRNAs related to its mechanisms and progression have been identified in diverse animal models and in human plasma or urine [17,24,25].

### 3.2. LncRNAs

LncRNAs are transcribed from intergenic, exonic, or distal protein-coding regions of the genome by RNA polymerase, followed by 3′-polyadenylation and 5′-end capping. LncRNAs are functional molecules that play various roles by interacting with mRNAs, miRNAs, DNAs, proteins, and small molecules. Another lncRNA promotes the miRNA-induced silencing complex (mi-RISC) to repress mRNA translation. Recently, lncRNAs have been increasingly recognized as important regulators in the pathophysiology of various kidney diseases and in kidney injury in both acute and chronic settings [26]. Several lncRNAs have been implicated in kidney fibrosis through fibrosis-related pathways, including transforming growth factor-β (TGF-β) signaling in experimental models. In addition, lncRNAs have been proposed to regulate diverse biological processes in kidney cells, including mesenchymal phenotype transition and vascular injury with microvascular rarefaction during kidney fibrosis [27]. Furthermore, some lncRNAs have been reported as potential prognostic biomarkers in patients with CKD [26].

### 3.3. CircRNAs

CircRNAs are generated from precursor mRNAs by back-splicing on a 5′ splice site to a 3′ splice site. Back-splicing arises from lariat-driven circularization, RNA-binding protein (RBP)- mediated circularization, intron-pairing-driven circularization, and intron circularization. By covalent joining on the 5′ and 3′ ends, the circular structure contributes to the stability against RNA degradation and de-adenylation, which gives a longer half-life than linear RNAs. Accumulating evidence has demonstrated the mechanisms of action of these circRNAs. Some circRNAs act as miRNA sponges and inhibit the activity of one or multiple miRNAs [1]. Growing evidence indicates that circRNAs contribute to kidney fibrosis and may represent promising therapeutic targets and biomarkers. Notably, circRNAs have been identified in kidney-derived exosomes and have been recommended as attractive candidates for non-invasive biomarker development in kidney diseases. Therefore, many researchers have reported that lncRNAs and circRNAs directly or indirectly regulate the alternative splicing of downstream target genes associated with kidney injury [21,28,29].

## 4. Mechanisms of Kidney Interstitial Fibrosis

Kidney fibrosis can develop in both the glomerular and tubulointerstitial compartments as a maladaptive wound-healing response, resulting in excessive deposition of ECM proteins [30,31]. Kidney interstitial fibrosis involves myofibroblast activation, excessive ECM accumulation, and the loss or dysfunction of renal tubular epithelial cells. Understanding the mechanisms behind interstitial fibrosis and developing effective interventions are crucial.

Resident interstitial fibroblasts and perivascular mesenchymal cells, located between the tubular basement membrane and peritubular capillaries, help maintain ECM homeostasis. Following injury, these cells proliferate and differentiate into ECM-producing myofibroblasts. Their activation is influenced by interactions among injured tubular epithelial cells, endothelial cells, pericytes, and infiltrating immune cells. The exact contributions of these cellular sources to the myofibroblast population are still debated in the literature [32,33,34,35,36].

In injured kidneys, such as those with ischemic, drug-, toxic-, or obstructive injuries, tubular epithelial cells may fail to complete adaptive repair. Instead, they can become “stuck” in the cell cycle and begin to release inflammatory and pro-fibrotic mediators [37]. These sustained signals activate nearby fibroblasts and attract immune cells, thereby promoting interstitial fibrosis. These processes propagate interstitial fibrosis through sustained myofibroblast activation, capillary rarefaction, and inflammatory signaling. In a murine unilateral ureteral obstruction (UUO) model, lineage-tracing studies suggested that the majority of myofibroblasts arise from resident interstitial mesenchymal cells, including fibroblasts and pericyte-related cells [32,38], EMT [39,40], EndMT [41], and MMT [42] have been implicated in kidney interstitial fibrogenesis. The relative contributions of interstitial myofibroblasts to fibrosis have not been clearly defined, but their transition processes are important for reshaping cell states and promoting pro-fibrotic signaling in injured tissues. Kidney interstitial fibrosis results from interactions among kidney cells, immune cells, and the extracellular matrix. We will discuss miRNAs that regulate fibroblast-to-myofibroblast activation, as well as MMT, EMT, and EndMT, and emphasize their roles in fibrotic remodeling.

## 5. Regulation of Myofibroblast Activation Derived from Resident Kidney Fibroblasts

Myofibroblast activation and subsequent ECM accumulation are central events in kidney interstitial fibrosis [5,6,30]. Activated myofibroblasts are the principal effector cells of fibrosis because they produce large amounts of ECM [7,31]. The acquisition of α-smooth muscle actin (α-SMA) expression is a hallmark of fibroblast-to-myofibroblast activation [5,7]. Although myofibroblasts are scarce in the normal kidney interstitium, their numbers markedly increase in fibrotic kidneys. Therefore, elucidation of the mechanisms underlying myofibroblast activation remains a key issue in kidney fibrosis [5,6,8]. Following kidney injury, injured tubular epithelial cells and infiltrating immune cells release a variety of pro-fibrotic mediators through autocrine and paracrine signaling, thereby promoting myofibroblast activation within the fibrotic microenvironment [5,6,8,31]. In the following section, we summarize the recent studies on miRNAs that regulate the activation of myofibroblasts derived from resident interstitial fibroblasts.

MiR-21 is one of the most extensively studied miRNAs involved in kidney fibrosis. Several studies have shown that miR-21 is upregulated and predominantly expressed in activated fibrotic regions, including tubulointerstitial fibroblasts, using in situ hybridization [43,44]. Glowacki et al. [43] reported that miR-21 is strongly upregulated in the kidneys of mice with UUO and in human kidneys with advanced fibrosis. Moreover, circulating miR-21 levels were higher in kidney transplant recipients with severe interstitial fibrosis/tubular atrophy (IF/TA) and were independently associated with IF/TA scores in multivariate linear regression analysis. In vitro, primary mouse kidney fibroblasts stimulated with TGF-β showed upregulation of miR-21 and actin alpha 2 (acta2), a myofibroblast marker [43]. Sun et al. [44] demonstrated that miR-21 is robustly upregulated in UUO kidneys and in TGF-β1-stimulated normal rat kidney-49F (NRK-49F) cells. MiR-21 mimics enhance the expression of collagen type I alpha 1 chain (Col1a1), fibronectin, and α-SMA, whereas miR-21a-5p antagonists and genetic deletion attenuate fibroblast activation and ameliorate UUO-induced interstitial fibrosis. Mechanistically, miR-21 directly targets Smad7, suppressing it and enhancing TGF-β/Smad3 signaling. Additionally, miR-21, programmed cell death protein 4 (PDCD4), and activation protein-1 (AP-1) form an autoregulatory loop that sustains AP-1–driven miR-21 expression and fibroblast activation [44]. PDCD4 is a tumor suppressor that negatively regulates AP-1 activity. AP-1 is a transcription factor complex that promotes pro-fibrotic gene expression [45]. Li et al. [46] showed that melatonin attenuates TGF-β1–induced fibroblast-to-myofibroblast transition in NRK-49F cells and UUO-induced kidney fibrosis. Melatonin reduces the expression of α-SMA, Col1a1, and fibronectin; inhibits phosphorylation of signal transducers and activators of transcription3 (STAT3); downregulates miR-21-5p level; and upregulates its antifibrotic targets of Sprouty1 (Spry1) and phosphatase and tensin homolog (PTEN). MiR-21-5p mimics or knockdown of Spry1 or PTEN partially reverses these protective effects, indicating that regulation of the miR-21-5p/PTEN and/or miR-21-5p/Spry1 axes plays a central role in fibrosis [46]. Zhao et al. [47] identified tubular epithelial cell–derived exosomes as a source of miR-21 fibrosis in UUO kidneys. TGF-β1-stimulated NRK-52E cells release miR-21–rich exosomes that were taken up by NRK-49F cells, leading to an increase in the α-SMA, collagen I, and fibronectin levels. Pharmacological inhibition of exosome release with GW4869 or genetic deletion of Rab27a reduces fibroblast activation and UUO-induced fibrosis. Moreover, miR-21–deficient exosomes preserve PTEN expression, suppress Akt activation, and ameliorate kidney interstitial fibrosis in vivo [47]. In a murine model of chronic kidney allograft dysfunction, renal miR-21a-5p expression was markedly upregulated, and therapeutic silencing of miR-21a-5p with a locked nucleic acid (LNA) inhibitor attenuated interstitial fibrosis, inflammatory cell infiltration, Banff lesion scores, and improved graft function. MiR-21a-5p expression in kidney fibroblasts is induced by macrophage-derived interleukin-6 (Il-6) via STAT3 activation. In addition, LPS-activated macrophages released small extracellular vesicles that are enriched in mature miR-21a-5p. These vesicles are taken up by kidney fibroblasts, which induce a profibrotic phenotype with increased Il-6 expression, connective tissue growth factor (CTGF), α-SMA, and collagens. Schauerte et al. [48] have identified Notch2 as a novel direct target of miR-21a-5p. Notch2 is a membrane-bound receptor that plays a critical role in kidney development. Consistent with previous reports showing that Notch2 signaling restrains the fibroblast-to-myofibroblast transition, Notch2 downregulation via miR-21a-5p upregulation promotes myofibroblast differentiation and activation [48].

MiR-26a-5p was significantly downregulated in human kidneys with interstitial nephritis and in UUO-injured mouse kidneys. Systemic infusion of a miR-26a-5p mimic after UUO surgery reduces kidney interstitial inflammation and fibrosis, lowers serum creatinine levels, and decreases TGF-β1 expression. In TGF-β1-stimulated NRK-49F cells, overexpression of miR-26a-5p diminishes fibroblast activation by lowering TGF-β receptor I and II levels, reducing Smad3 phosphorylation, and decreasing the expression of NF-κB, matrix metalloproteinase-9, and mesenchymal markers such as vimentin, fibronectin, and α-SMA, thereby limiting the transition from fibroblast to myofibroblast [49].

Both miR-29 and miR-30 families are widely recognized as anti-fibrotic regulators of kidney interstitial fibrosis. Their expression is consistently reduced in fibrotic human kidneys and murine models of UUO, angiotensin II (Ang II) infusion, and ischemic reperfusion injury (IRI). In these models, overexpression of miR-29 or miR-30 through mimic- or vector-mediated restoration suppressed fibroblast activation and expression of myofibroblast markers, limited extracellular matrix accumulation, and attenuated interstitial fibrosis [50,51,52,53,54].

In mice with UUO, kidney expression of p53 and miR-34a was upregulated. Transfection of a miR-34a mimic into NRK-49F cells increases the expression of Col1a1, Col1a2, and Tgf-β1 and robustly induces α-SMA. Inhibition of miR-34a reduces the expression of TGF-β1-induced Acta2. Importantly, miR-34a does not activate the TGF-β/Smad pathway, and TGF-β1 does not increase miR-34a expression. Thus, miR-34a-induced α-SMA upregulation occurs independently of canonical TGF-β/Smad signaling, indicating that the p53/miR-34a axis promotes fibroblast-to-myofibroblast differentiation and contributes to kidney interstitial fibrosis [55].

Sakuma et al. [56] generated mice with Dicer deletion in platelet-derived growth factor receptor (pdgfr)-β–positive mesenchymal cells and showed that loss of Dicer exacerbates UUO- and folic acid-induced interstitial fibrosis, accompanied by pdgfr-β overexpression and miR-9-5p downregulation. In primary kidney fibroblasts, inhibition of miR-9-5p further increased PDGFRb and Acta2 under TGF-β1 stimulation. These findings indicate that miR-9-5p restrains pdgfr-β–driven myofibroblast differentiation and may function as an antifibrotic regulator in kidney interstitial fibrosis [56]. Table 1 summarizes other recent studies supporting the role of miRNAs in fibroblast activation.

## 6. Regulation of Epithelial–Mesenchymal Transition (EMT)

EMT is a phenotypic process in which epithelial cells lose their epithelial characteristics and acquire mesenchymal features [63]. EMT can be summarized as follows: (1) dissolution of epithelial cell junctions and loss of cell polarity, (2) downregulation of epithelial gene expression, (3) reorganization of the cytoskeletal architecture and changes in cellular morphology, and (4) acquisition of the ability to secrete factors that remodel the extracellular matrix. Classical EMT is defined as fully convert and migrate tubular epithelial cells into myofibroblasts in kidney interstitium, whereas partial EMT is defined that tubular epithelial cells remain attached to the basement membrane, relay profibrotic signals to kidney interstitium, promote myofibroblast activation, and sustain inflammation, which has also been recognized as a key driver of interstitium fibrosis [40,64,65]. As recent lineage-tracing approaches have directly demonstrated that trans-differentiation of tubular epithelial cells into myofibroblasts and migration are relatively rare, basic research migration is relatively rare, basic research evidence suggests that the more regenerative fibrotic process is considered to be partial EMT rather than classical EMT. In the following section, we review representative reports on miRNAs involved in EMT-related processes in kidney fibrosis, including but not limited to partial EMT.

The expression of miR-17 was upregulated in the serum of patients with diabetic nephropathy (DN) and in both the serum and kidney tissues of db/db mice. Systemic administration of an LNA–miR-17 inhibitor attenuated kidney fibrotic changes in db/db mice, as determined by the histological assessment of PAS and Masson staining. In vitro, TGF-β1–induced HK-2 cells (and human mesangial cells) increased the expression of miR-17, whereas miR-17 inhibition increased the levels of E-cadherin and decreased those of vimentin, fibronectin, and collagen I. Mechanistically, Smad7 was identified as a direct target of miR-17 by dual-luciferase reporter assays, and the inhibition of miR-17 resulted in upregulated Smad7 expression, suggesting that miR-17 promotes TGF-β1–driven fibrotic/EMT-like responses through Smad7 suppression [66].

MiR-19 is also associated with renal tubular EMT. miR-19 expression is upregulated in peripheral blood from patients with kidney fibrosis and in UUO kidneys and is also induced by TGF-β1 in NRK-52E cells. MiR-19 suppresses PTEN by binding its 3′UTR, followed by activating Akt signaling, decreasing E-cadherin expression, and increasing α-SMA/fibronectin expression. miR-19 inhibition restores PTEN and blunts TGF-β1–induced EMT. In mice with UUO, tail vein miR-19 agomir increased collagen deposition in Masson’s and Sirius Red staining, whereas miR-19 antagomir alleviated interstitial fibrosis [67]. Beyond miR-19, multiple EMT-linked miRNAs converge on the PTEN/AKT axis, including miR-21 in UUO kidneys treated with pure total flavonoids from Smilax glabra (PTFS) and miR-382 in aristolochic acid (AA)–induced kidney injury model [68,69]. The miR-21 family is also an important factor in the EMT during kidney interstitial fibrosis. In rats with UUO, PTFS suppressed EMT and mitigated kidney interstitial fibrosis, as assessed by real-time polymerase chain reaction (PCR) and Western blot, with reduced α-SMA expression and restored E-cadherin expression. PTFS also modulated the miR-21/PTEN/PI3K–Akt axis in TGF-β1-stimulated HK-2 cells. Additionally, overexpression of miR-21 reduced the expression of PTEN and E-cadherin and promoted α-SMA and phosphorylation of PI3K/Akt, whereas inhibition of miR-21 produced the opposite phenotype, indicating that blockade of miR-21-mediated PTEN suppression is a key anti-EMT mechanism in UUO-induced fibrosis [68]. In aristolochic acid (AA)-induced nephropathy, the kidney expression of miR-382 increases during the transition from AKI to CKD, along with progressive tubulointerstitial fibrosis. Genetic deletion or pharmacological inhibition of miR-382 partially reversed inflammatory and fibrotic responses and reduced EMT-like changes. Mechanistically, miR-382 directly targets the 3′UTR of PTEN through luciferase assays, resulting in PTEN loss and subsequent activation of AKT signaling. In tubular epithelial cells, inhibiting PTEN and/or overexpressing miR-382 worsened the loss of epithelial markers and the increase in mesenchymal markers, supporting the PTEN/AKT-dependent EMT pathway. NF-κB functions upstream, as NF-κB siRNA decreased AA-induced miR-382 upregulation, indicating an NF-κB–dependent miR-382/PTEN/AKT cascade in tubular injury [69].

The expression of miR-23a is upregulated in kidney tissues of patients with DN and in HK-2 cells exposed to HG, whereas the Ski-related novel protein N (SnoN), known as a critical negative regulator of the TGF-β/Smad signal pathway, is downregulated in diabetic kidneys among patients and HG-exposed HK-2 cells. Silencing miR-23a restores SnoN expression and attenuates HG-induced EMT and fibrogenic responses. Conversely, miR-23a overexpression suppresses SnoN expression and aggravates EMT and ECM production. Additionally, overexpression of SnoN inhibits miR-23a, whereas SnoN knockdown partially reverses this protection, supporting that the miR-23a/SnoN axis drives tubular EMT and kidney fibrogenesis in vitro [70]. Similarly, miR-130a-3p has also been reported to promote TGF-β1—induced EMT and fibrotic responses in tubular epithelial cells by directly targeting SnoN. Inhibition of miR-130a-3p reduces p-Smad2/3, increases Smad7, and attenuates EMT markers by modulating SnoN in HK-2 and primary human renal proximal tubular epithelial cells [71].

Members of the miR-27 family exert both pro- and anti-fibrotic effects. In db/db mice and HG-treated HK-2 cells, miR-27a-3p expression was upregulated, whereas that of prohibitin and transmembrane BAX inhibitor motif-containing 6 (TMBIM6), which are direct miR-27a-3p targets, was downregulated. In a previous study, prohibitin, a multifunctional protein, was ubiquitously present in multiple cellular compartments. TMBIM6 ameliorates IRI-induced AKI by regulating mitochondrial homeostasis [72]. Silencing of miR-27a-3p attenuates kidney interstitial fibrosis, accompanied by reduced profibrotic factors and restoration of E-cadherin expression. Anti-miR-27a-3p also alleviates mitochondrial dysfunction and endoplasmic reticulum (ER) stress signaling, thereby limiting apoptosis and matrix accumulation. In HG-treated HK-2 cells, restoring prohibitin or TMBIM6 recapitulated the protective effects of miR-27a-3p inhibition, supporting miR-27a-3p/prohibitin or TMBIM6 axis in diabetic tubulointerstitial injury [73]. In STZ-induced DN of rats and HG-stimulated NRK-52E cells, miR-27a is increased in parallel with kidney fibrosis and activation of Wnt/β-catenin signaling, including β-catenin nuclear translocation. Mechanistically, miR-27a directly targets the 3′UTR of secreted frizzled-related protein 1 (Sfrp1), known as a Wnt antagonist by luciferase assays. Functionally, inhibition of miR-27a decreases collagen IV and α-SMA levels and restores E-cadherin. Conversely, Sfrp1 knockdown aggravated EMT-like changes, whereas miR-27a inhibition partially reversed these effects. Collectively, these findings support a pro-fibrotic axis of miR-27a/Sfrp1/Wnt–β-catenin in diabetic kidney injury [74]. By contrast, miR-27b-3p expression was downregulated in UUO kidneys and in TGF-β1–treated HK-2 cells. Restoration of miR-27b-3p suppresses TGF-β1–induced EMT and apoptosis, concomitant with a reduction in α-SMA, collagen III, fibronectin, and vimentin. STAT1 was identified as a direct target of miR-27b-3p using dual-luciferase assays, and its overexpression counteracted the anti-apoptotic effects of miR-27b-3p. In vivo, miR-27b-3p overexpression alleviated UUO-associated tubulointerstitial injury and fibrosis, supporting that miR-27b-3p is an anti-fibrotic EMT-limiting regulator [75].

Recently, several studies have demonstrated that the modulation of lncRNA activity in the kidney is an important mechanism in EMT. The upregulation of lncRNA H19 alleviated interstitial fibrosis and EMT, accompanied by a reduction in lipid deposition and inflammatory responses. LncRNA H19 is capable of increasing ACSL1 levels by sponging miR-130a-3p, leading to the suppression of EMT, inflammatory responses, and prevention of interstitial fibrosis [76].

Other recent studies supporting the role of miRNAs in the EMT are summarized in Table 2.

## 7. Regulation of Endothelial-Mesenchymal Transition (EndMT)

EndMT is a phenotypic program in which endothelial cells lose endothelial features and acquire mesenchymal characteristics and has been implicated in kidney injury and fibrosis [41]. In injured kidney capillaries, endothelial dysfunction enhances local inflammation, ECM deposition, and capillary rarefaction, thereby promoting tubulointerstitial fibrosis [112]. During EndMT, endothelial cells pass through an intermediate state with co-expression of endothelial and mesenchymal markers, accompanied by increased expression of mesenchymal markers (e.g., α-SMA, collagen I, fibronectin, and vimentin) and reduced expression of endothelial markers (e.g., von Willebrand Factor (vWF), CD31, and Vascular Endothelial (VE)-cadherin) [41]. TGF-β signaling drives EndMT through canonical Smad-dependent pathways and non-canonical pathways, such as MAPK and PI3K/Akt [41]. Noncoding RNAs, including miRNAs, are recognized as regulators of endothelial dysfunction [113]. Here, we summarize the relationship between EndMT and miRNAs.

In an in vitro study, TGF-β1 stimulation of human umbilical vein endothelial cells (HUVECs) reduced VE-cadherin expression and increased the mesenchymal marker SM22α. During this process, HUVECs displayed cellular hypertrophy, lost sprouting capacity, and downregulated miR-20a. MiR-20a targeted TGF-β receptors (ALK5, TGF-βR2) and SARA, which are crucial for TGF-β signaling. Additionally, FGF2 increased miR-20a expression and inhibited EndMT in TGFβ1-stimulated endothelial cells. HUVECs transfected with miR-20a mimics blunted TGF-β1 stimulation, while anti-miR-20a abrogated FGF2′s protective effect against TGF-β1-induced EndMT. These results indicate that FGF2 inhibits TGF-β signaling by upregulating miR-20a during EndMT [114].

Huang et al. [51] found that the expression of miR-29a-3p in fibrotic mouse kidneys was reduced after unilateral IRI. Intravenous delivery of human umbilical cord mesenchymal stem cell-derived exosomes containing high levels of miR-29a-3p improved microvascular integrity, reduced vascular rarefaction, and attenuated interstitial fibrosis. This benefit was supported by miR-29a-3p gain and loss of experiments and was linked to the direct targeting of COL1A1 in fibroblasts and TNFR1 in endothelial cells [51].

Wang et al. [115] found that elevated parathyroid hormone (PTH) levels drive EndMT and calcific remodeling in CKD rats induced by 5/6 nephrectomy and a high-phosphate diet. In vitro and in vivo studies showed that endothelial miR-29a-5p is significantly reduced and targets γ-secretase-activating protein (GSAP). Overexpression of miR-29a-5p inhibits PTH-induced EndMT, and the use of miR-29a-5p mimics or γ-secretase inhibitors blocks the Notch1 pathway, preventing EndMT. PTH induces valvular EndMT through the miR-29a-5p/GSAP/Notch1 pathway [115].

miR-126-3p was detected in renal endothelial cells by in situ hybridization and was reduced in fibrotic kidneys after UUO. In HUVECs, EndMT induced by TGF-β2 plus Il-1β was associated with a reduction in miR-126-3p levels. Transfection with a miR-126-3p mimic preserved CD31 expression and reduced fibronectin levels, supporting partial protection against EndMT, although it did not restore vWF or VE-cadherin levels. These findings suggest that the loss of miR-126-3p accompanies endothelial phenotypic instability and that replenishment may help maintain endothelial features [116].

Qian et al. [117] found that lncRNA taurine-upregulated gene 1 (TUG1) is upregulated in UUO kidneys and hypoxia-injured HUVECs. Silencing of TUG1 reduced kidney fibrosis, restored vascular endothelial growth factor (VEGF) levels, and lowered α-SMA, TGF-β1, and HIF-1α expression. It also preserved CD31-positive endothelium, reduced CD31- and αSMA-double-positive EndMT-phenotypic cells, and improved peritubular capillary perfusion on fluorescence microangiography. Mechanistically, cytoplasmic TUG1 directly bound miR-542-3p and miR-542-3p also bound HIF-1α using luciferase assays and bioinformatics analysis. TUG1 knockdown increased miR-542-3p and inhibition of miR-542-3p reversed the protection, supporting a miR-542-3p/HIF-1α/VEGF axis [117].

## 8. Regulation of Macrophage–Mesenchymal Transition

Macrophages adopt different phenotypes and play crucial roles in tissue homeostasis in both normal and fibrotic kidneys. They are often classified as pro-inflammatory (M1) or anti-inflammatory (M2) macrophages [118]. M1 macrophages contribute to the host defense and aggravate kidney injury, whereas M2 macrophages are associated with injury resolution and repair. However, in later stages of kidney injury, M2 macrophages can also produce pro-fibrotic mediators, including Il-10 and TGF-β1, thereby contributing to kidney interstitial fibrosis [118,119]. Prolonged pro-fibrotic activation has been linked to macrophage-to-myofibroblast transitions (MMTs), in which macrophages acquire myofibroblast-like features and contribute as matrix-producing cells during kidney fibrosis [119,120]. Because MMT is considered an important mechanism and a potential therapeutic target, we discuss the miRNA regulation of MMT in kidney interstitial fibrosis in the following section [119,121].

Biopsy specimens from patients with chronic active kidney graft rejection showed CD68/α-SMA double-positive cells consistent with MMT, and IRI-treated mice likewise exhibited numerous F4/80/α-SMA double-positive cells in the kidney interstitium. After IRI, the mouse kidneys displayed tubular senescence markers (p21/p16). In addition, paclitaxel-treated senescent HK-2 cells released extracellular vesicles enriched with miR-20a-5p and miR-21-5p. These miRNAs drive M2-like polarization and expression of fibrotic markers in macrophages. These miRNAs mechanistically suppress Smad7, increase phosphorylation of Smad3, and form a positive feedback loop with TGF-β1. Mimic transfection in macrophages recapitulated the EV-driven phenotype by increasing M2-associated features, elevating TGF-β1, and upregulating myofibroblast markers, whereas miR inhibitors attenuated these pro-fibrotic markers and partially blunted the effects induced by senescent HK-2 cells-derived EVs [122].

In LPS-stimulated RAW264.7 cells, miR-92a-3p overexpression augmented M1-associated inflammatory responses with increases in iNOS, Il-6, and TNF/TNF-α, whereas miR-92a-3p inhibition blunted these changes. Mechanistically, LIN28A was validated as a direct target of miR-92a-3p, and LIN28A overexpression rescued the pro-inflammatory effects of miR-92a-3p. LIN28 is known for its selective regulation of mRNA expression and influences cell proliferation, differentiation, and metabolic processes across various cell types [123]. In vivo, systemic administration of a miR-92a-3p inhibitor attenuated UUO-induced kidney inflammation and interstitial fibrosis, accompanied by restored LIN28A expression and reduced iNOS and α-SMA expression. While MMT was not directly examined, miR-92a-3p was shown to amplify the M1-type inflammatory activation of macrophages by targeting LIN28A and worsen kidney inflammation and fibrosis in vivo [124].

In a mouse model of AA-induced nephropathy, miR-382 was found to be enriched in kidney macrophages isolated by flow cytometry. Systemic and macrophage-specific genetic deletion of miR-382 reduced CD206 + M2-like polarization and attenuated interstitial fibrosis, whereas miR-382 overexpression in bone marrow-derived macrophages aggravated kidney injury and fibrosis. Mechanistically, miR-382 directly targeted signal regulatory protein-alpha (SIRP-α), thereby enhancing STAT3 phosphorylation and promoting a pro-fibrotic macrophage program. These findings highlight a miRNA-controlled macrophage axis that can amplify pro-fibrotic signaling and tubular injury in kidney fibrosis [125].

Kidney miR-150 levels increased in the FA–induced AKI-to-CKD model and in HK-2 cells co-cultured with macrophages. This upregulation was accompanied by reduced suppressor of cytokine signal 1 (SOCS1) expression and increased levels of α-SMA, fibronectin, and collagen I. SOCS1 is an antifibrotic effector protein [126]. Systemic administration of LNA–anti-miR-150 restored SOCS1 expression, reduced kidney IFN-γ/Il-6/TNF-α expression, diminished macrophage accumulation, and attenuated tubulointerstitial fibrosis. In vitro co-culture with macrophages increased miR-150 expression in HK-2 cells. This was accompanied by reduced SOCS1, activation of the JAK/STAT pathway, and upregulation of pro-fibrotic markers. These changes were partially reversed upon treatment with a miR-150 antagonist. Although MMT was not directly assessed, the data highlighted miRNA-driven macrophage–tubular crosstalk that may create a pro-fibrotic milieu permissive to MMT [127].

## 9. Regulation of Pericyte-to-Myofibroblast Transition

Pericytes are perivascular cells located on the abluminal surface of capillary endothelial cells. Under physiological conditions, they stabilize the microvascular network and maintain vascular homeostasis through angiogenic growth factors [128]. During kidney injury, endothelial cell–derived signals, including VEGF, TGF-β1, and PDGF, can activate neighboring pericytes [42,129]. Activated pericytes detach from endothelial cells, lose their vessel-stabilizing functions, migrate into the interstitial space, and transition into myofibroblasts [129]. This pericyte-to-myofibroblast transition contributes to peritubular capillary rarefaction and promotes interstitial fibrosis [42,129].

A recent study explored the miRNA-dependent regulation of pericyte-to-mesenchymal transition in kidney interstitial fibrosis using primary kidney pericytes. Hu et al. [130] found that bone marrow mesenchymal stem cell–derived exosomes delivered miR-34c-5p to TGF-β1-stimulated primary renal pericytes, suppressing α1,6-fucosyltransferase (FUT8) and reducing core fucosylation of pro-fibrotic receptors. This inhibition decreased pro-fibrotic signaling and extracellular matrix production in vitro and improved kidney interstitial fibrosis in vivo. The transfer of miR-34c-5p was driven by the CD81–EGFR complex, with similar effects observed in kidney fibroblasts and macrophages [130].

**Figure 1:**
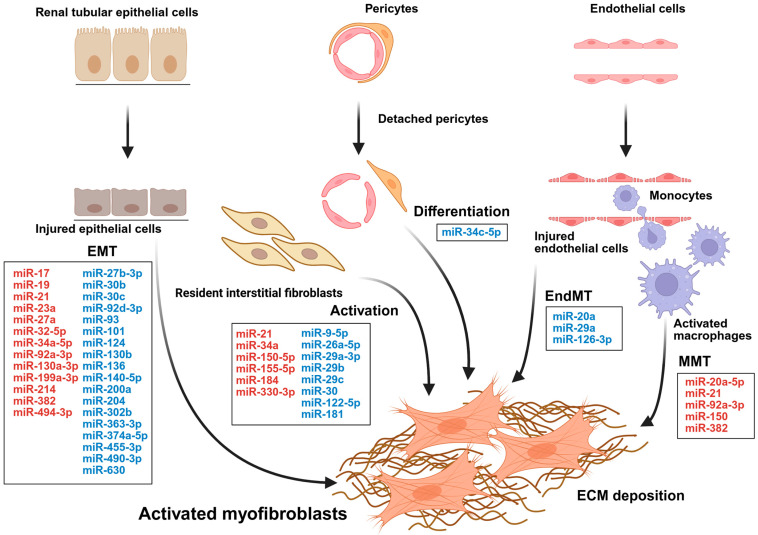
Myofibroblast activation in kidney interstitial fibrosis. Resident interstitial fibroblasts, pericytes, injured renal tubular epithelial cells undergoing epithelial-to-mesenchymal transition (EMT), endothelial cells undergoing endothelial-to-mesenchymal transition (EndMT), and activated macrophages derived from circulating monocytes undergoing macrophage-to-mesenchymal transition (MMT) are all known sources of myofibroblasts in fibrotic processes. Various miRNAs have been implicated in these processes. Some miRNAs exert protective effects (shown in blue), whereas others directly promote kidney interstitial fibrosis (shown in red).

In summary, Figure 1 illustrates key miRNAs and their associated regulatory pathways that contribute to mesenchymal transition during kidney interstitial fibrosis.

## 10. Clinical Utilities of MiRNAs in Acute Kidney Injury and Chronic Kidney Disease

### 10.1. IgA Nephropathy (IgAN)

IgAN is the most prevalent form of primary glomerulonephritis and predominantly affects children and young adults. The most striking feature of IgAN is the mesangial deposition of IgA or IgA-containing immune complexes [131,132]. Pathological risk stratification is commonly guided by the Oxford classification (MEST-C), in which the T-score reflects the extent of tubular atrophy/interstitial fibrosis (TA/IF), T0 (0–25%), T1 (26–50%), and T2 (>50%) [133]. Notably, TA/IF represents one of the strongest predictors of long-term kidney outcome across cohorts, underscoring the clinical importance of interstitial fibrotic remodeling in IgAN [134,135]. However, the mechanisms driving tubulointerstitial fibrosis and optimal therapeutic strategies remain unclear. Recent studies have revealed that ncRNAs regulate inflammation-fibrotic programs; therefore, we summarize recent evidence on ncRNA-associated pathways relevant to interstitial fibrosis in IgAN.

Fang et al. [136] reported that miR-382 promotes kidney tubulointerstitial fibrosis by targeting heat shock protein 60 (HSPD1), which is accompanied by decreased thioredoxin (Trx) expression and increased oxidative stress (e.g., elevated 3-nitrotyrosine [3-NT]). In kidney biopsy specimens from IgA nephropathy patients with moderate-to-severe tubulointerstitial fibrosis, HSPD1 and Trx were reduced, whereas 3-NT was increased, supporting the clinical relevance of this pathway [136].

Duan et al. [137] showed that miR-185-5p directly targets tight junction protein 1 (TJP1) in tubular epithelial cells. miR-185-5p-mediated TJP1 suppression promoted a profibrotic phenotype in HK-2 cells, with increased α-SMA, fibronectin, and collagens. Clinically, urinary miR-185-5p levels were significantly higher in IgAN patients with Oxford T1–T2 lesions than in those with T0, supporting its potential utility as a noninvasive marker of tubulointerstitial injury [137].

Szeto et al. [138] reported that intrarenal miR-21 expression was significantly increased in IgAN compared with hypertensive nephrosclerosis and showed a modest correlation with the severity of tubulointerstitial fibrosis. Higher intrarenal miR-21 levels were associated with poorer kidney event-free survival in univariate analysis, although the association became borderline after adjustment for histologic scarring. These findings suggested that intrarenal miR-21 expression was increased in patients with IgAN, modestly correlated with the severity of histologic damage, and predictive of subsequent kidney function loss [138].

### 10.2. Diabetic Nephropathy (DN)

DN is the leading cause of CKD and ESKD [139,140]. Recent data show that approximately 529 million people worldwide will be affected by diabetes in 2021, and it is predicted that the number will increase to 1.3 billion by 2050 [141,142]. Parallel to the increasing prevalence of diabetes, the global incidence of DN has markedly increased. In fact, around 30–40% of people with type 1 or type 2 diabetes develop DN [143].

Hyperglycemic conditions induce the glycation of proteins and lipids, the formation of advanced glycation end products, and activated polyol pathway activity, leading to glomerular and tubular injury [144]. Moreover, hyperglycemia triggers the production of proinflammatory mediators such as TNF-α, IL-1, and IL-6, leading to endothelial dysfunction and tubulointerstitial fibrosis. DN involves a complex interplay of metabolic changes, hemodynamic alterations, and inflammation. Pathological features of DN include mesangial expansion, glomerular hypertrophy, tubulointerstitial fibrosis from ECM protein accumulation, podocyte dysfunction, and basement membrane thickening [144].

Activation of TGF-β/Smad signaling is a key mechanism in kidney fibrosis, influencing inflammation, oxidative stress, endothelial dysfunction, and podocyte injury, which leads to proteinuria in DN [21,145]. In addition, hyperglycemic condition-mediated reactive oxygen species (ROS) lead to cellular injury, endothelial dysfunction, and activation of pro-inflammatory signaling pathways, such as NF-κB and JAK/STAT, all of which contribute to mesangial expansion, glomerulosclerosis, and tubulointerstitial fibrosis [145].

Recently, several studies have demonstrated that modulation of ncRNA activity in the kidney is an important mechanism underlying DN pathogenesis.

Ding et al. reported that miR-10a and -10b are downregulated in the kidneys of STZ-treated DN mice and patients with DN, as well as in HG-treated human glomerular podocytes and tubular epithelial cells [146]. In another experiment [147], miR-10a/10b overexpression reduces kidney fibrosis in STZ-treated diabetic mice by inhibiting TGF-β/Smad signaling, a process reversed by anti-miR-10a and -10b. Luciferase reporter analysis in human glomerular podocytes and tubular epithelial cells revealed that miR-10a and miR-10b target the 3′UTR of TGFBR1, which regulates fibronectin and α-SMA. In summary, miR-10a/b modulates kidney fibrosis by directly targeting TGFBR1 and influencing TGF-β/Smad signaling.

In a study by Kim et al. [148], it was found that renal miR-144-3p regulates TGF-β1-induced oxidative stress and fibrosis. This miRNA is upregulated in the urine and kidneys of spontaneously hypertensive rats (SHRs) and STZ-treated SHRs. A luciferase reporter assay showed that miR-144-3p binds to the 3′UTR of NRF2, a negative regulator of oxidative stress linked to metabolism and inflammation. TGF-β1 suppresses NRF2 and increases ROS, leading to kidney fibrosis via upregulation of miR-144-3p. These findings suggest that targeting the miR-144-3p/NRF2 pathway may offer therapeutic potential for CKD in the context of hypertension and diabetes [148].

Another type of ncRNA, circRNA, has been reported to play an important role in the regulation of kidney fibrosis in DN. Jiang et al. [149] demonstrated that circ_0054633 was upregulated more in the serum of patients with DN than in those without DN, which was positively correlated with the kidney fibrotic area. Attenuating upregulation of circ_005463 protected HG-treated HRMCs from cell proliferation and ECM accumulation via downregulation of TGF-β1/SMAD3 signaling. To investigate the underlying mechanism, they co-transfected with luciferase reporter vectors containing the circ_0054633 or circ_0054633 mutant and miR-NC or miR-136-5p mimic. These results have demonstrated the role of circ_0054633 as a sponge for miR-136-5p. In addition, the experiment showed that miR-136-5p bound to the 3′UTR of SMAD3 in HRMCs. Furthermore, silencing of circ_0054633 in db/db mice attenuated collagen IV and fibronectin activation induced by TGF-β1/SMAD3 signaling, via regulation of miR-136-5p/SMAD3 signaling. In summary, this study suggests that circ_0054633 plays a clinically important role in regulating kidney fibrosis via miR-136-5p/SMAD3 [149].

LncRNAs also have functional roles in interactions with small molecules, such as mRNAs, miRNAs, or proteins. Wang et al. [150] identified the role of lncRNA small nucleolar RNA host gene 14 (SNHG14) in DN. SNHG14 expression was elevated in the kidneys of STZ-induced DN mice and in HG-treated HRMCs. Next, they confirmed that SNHG14 silencing inhibited cell proliferation and fibrosis in HG-treated HRMCs and kidneys of DN mice. Luciferase reporter experiments demonstrated that SNHG14 bound to miR-30e-5p in HRMCs, and miR-30e-5p also bound to the 3′UTR of SRY-box transcription factor 4 (SOX4) expression in HRMCs. SOX4 is a critical factor involved in tubular epithelial cell (TEC) dedifferentiation and fibroblast activation [151]. In summary, SNHG14 reduces cell proliferation and fibrotic phenotype in DN via the miR-30e-5p/SOX4 axis [150].

Recent studies on the association between ncRNAs and fibrosis activation in DN are summarized in Table 3.

### 10.3. Hypertensive Nephropathy

Hypertensive nephropathy refers to chronic kidney damage caused by prolonged high blood pressure. It is the leading cause of CKD and ESKD [163]. Pathologically, hypertensive nephropathy is characterized by arterial and arteriolar sclerosis, glomerulosclerosis, and tubulointerstitial fibrosis, which together result in the progressive loss of kidney function. In recent years, non-coding RNAs, including miRNAs and lncRNAs, have emerged as important regulators of gene expression in kidney disease. Given their central roles in gene regulation, miRNAs and lncRNAs have been intensively studied for their contribution to the pathogenesis of hypertensive nephropathy and their potential as diagnostic biomarkers or therapeutic targets.

Several miRNAs are associated with kidney injury in patients with chronic hypertension. Broad miRNA expression profiles in hypertensive models and patient samples reveal a dysregulated miRNA signature that correlates with kidney tissue damage and functional decline. In a biopsy-based study of patients with hypertensive nephrosclerosis, Wang et al. reported increased intrarenal expression of several miRNAs, including members of the miR-200 family (miR-200a, miR-200b, miR-141, and miR-429) as well as miR-205 and miR-192, compared to controls [164]. In that study, higher intrarenal levels of these miRNAs correlated with the degree of proteinuria, and miR-200a and miR-205 showed inverse correlations with the estimated glomerular filtration rate. The miR-200 family regulates epithelial–mesenchymal transition (EMT) by targeting the transcription factor ZEB1/2. Consistent with this regulatory axis, ZEB1 expression was inversely correlated with miR-429, and ZEB2 expression was inversely correlated with miR-200a, miR-200b, and miR-429 in biopsy specimens. Taken together, these observations support an association between intrarenal miRNA dysregulation and clinical/pathological severity in hypertensive nephrosclerosis; however, the cross-sectional nature of the data limits causal inference regarding whether these miRNAs are protective responses or contributors to injury [164].

Another miRNA linked to kidney fibrosis in hypertension is miR-21. MiR-21 is a well-known pro-fibrotic miRNA found in many organs. In a mouse model of deoxycorticosterone acetate (DOCA)-salt hypertension, which induces high blood pressure and kidney injury, miR-21 was among the most upregulated miRNAs in injured kidneys [165]. Notably, urinary miR-21 (normalized to creatinine) levels rose early after DOCA-salt treatment (detectable by day 4 in their analysis) and were suggested to increase before albuminuria, indicating its potential as an early marker of hypertensive kidney injury in that model [73]. The same study also reported an increased expression of additional miRNAs (including miR-146b, miR-155, and miR-132) in the kidney during DOCA-salt exposure [165].

Conversely, some miRNAs appear to play protective roles by blunting the impact of hypertension on the kidneys. One striking example is miR-204-5p, which is highly enriched in the kidney tissue [166]. In patients with hypertension or hypertensive nephrosclerosis, levels of miR-204-5p in the kidneys are significantly lower than in normotensive controls. This downregulation is mirrored in animal models, such as hypertensive Dahl salt-sensitive rats and Ang II-induced hypertensive mice, which show reduced renal miR-204-5p levels. Knocking out the mir-204-5p gene in mice exacerbates albuminuria, interstitial fibrosis, and arteriolar thickening. Additionally, inhibiting miR-204-5p in hypertensive rats worsens kidney artery sclerosis and fibrosis. MiR-204-5p normally targets SHP2 mRNA; its loss results in SHP2 upregulation and overactivation of the STAT3 pathway, leading to inflammation and fibrosis. Thus, miR-204-5p serves as a protective mechanism against hypertension-related kidney injury by regulating SHP2/STAT3 signaling.

One of the best-characterized lncRNAs is TUG1, which has emerged as a promoter of Ang II-mediated kidney injury. A recent study showed that TUG1 expression is significantly increased in renal tubular epithelial cells following Ang II treatment [167]. In vivo, mouse models of Ang II-induced hypertension and fibrosis exhibit upregulation of TUG1 in the kidneys, particularly in areas of tubulointerstitial fibrosis. Tug1 expression is higher in human kidney biopsies with fibrotic lesions than in non-fibrotic samples. It interacts with the mineralocorticoid receptor (MR) and acts as a competing endogenous RNA, sequestering miR-29b-3p, an anti-fibrotic miRNA. This interaction promotes fibrotic responses by relieving the repression of genes involved in extracellular matrix formation. Reducing TUG1 expression reduced angiotensin II-induced profibrotic gene expression and kidney fibrosis markers, whereas increased TUG1 expression worsened fibrosis. These findings highlight TUG1’s role in linking renin–angiotensin–aldosterone system signaling to pro-fibrotic pathways in hypertensive kidney disease [167].

**Figure 2:**
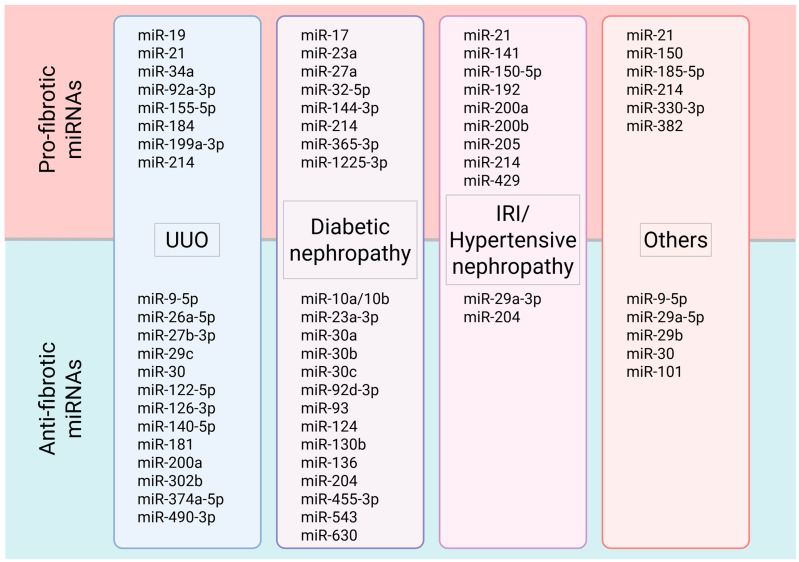
List of pro-fibrotic (upregulated) and anti-fibrotic (downregulated) miRNAs in several kidney fibrosis models and clinical settings, including unilateral ureteral obstruction (UUO), diabetic nephropathy, ischemia–reperfusion injury (IRI) or hypertensive nephropathy, and other etiologies, across rodent and human studies. UUO, unilateral ureteral obstruction; IRI, ischemia–reperfusion injury.

**Figure 3:**
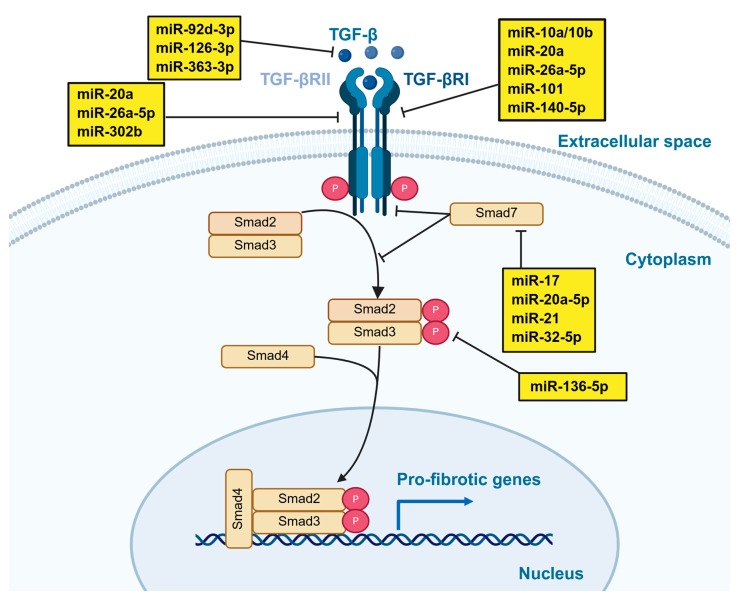
Regulation of miRNAs in TGF-β/Smad-mediated kidney interstitial fibrosis. In the canonical TGF-β/Smad pathway, the expression of multiple miRNAs is regulated, and these miRNAs are implicated in kidney interstitial fibrosis. Normal arrows indicate activation and nuclear translocation of Smad proteins. T-bar arrows indicate inhibition of the indicated proteins. TGF-β, transforming growth factor-β; TGF-βRI/II, transforming growth factor-β receptor type I/II; Smad2/3/4/7, mothers against decapentaplegic homolog 2/3/4/7.

In summary, Figure 2 presents key miRNAs identified across experimental models of kidney fibrosis in both rodent and human studies. Additionally, within the TGF-β/Smad pathway, which is essential for kidney interstitial fibrosis, several miRNAs associated with this pathway are summarized in Figure 3.

## 11. Therapeutic Potential and Limitations for MicroRNA-Based Therapies

Although circulating miRNAs have attracted interest as non-invasive biomarkers in kidney-related diseases, evidence that specifically links serum or plasma microRNAs to kidney interstitial fibrosis remains relatively limited [22]. Nevertheless, several studies in the field of AKI, such as cardiac surgery-associated and sepsis-associated AKI, have detected specific miRNAs in serum, plasma, or urine samples and have related their levels to disease severity and clinical outcomes. Similar studies have also been reported in patients with DN or IgAN using serum, plasma, or urine samples. Among these candidates, miR-21 has been repeatedly reported across multiple kidney diseases and is increasingly recognized as a frequently investigated biomarker.

Lademirsen (RG-012, SAR339375; Regulus Therapeutics/Sanofi) is an investigational anti-miR-21 oligonucleotide. Based on robust anti-fibrotic and nephroprotective effects of anti-miR-21 oligonucleotides in preclinical Alport models [168], a phase 2 trial (HERA; NCT02855268) was conducted in adults with Alport syndrome at risk of rapid progression [169]. However, the HERA study was terminated after an interim futility analysis, and no significant differences were observed between lademirsen and placebo in eGFR at any time point. For patients with autosomal dominant polycystic kidney disease (ADPKD), miR-17 has been identified as critically involved in ADPKD development [170,171]. The anti-miR-17 oligonucleotides (RGLS4326; next-gen RGLS8429) reduce cytogenesis in mouse models and have progressed to early clinical testing in ADPKD. Although miRNA-targeted therapies have not yet demonstrated clear clinical efficacy in human kidney diseases, this trial remains highly informative as an early attempt to translate miRNA-targeted therapeutics from animal models to human kidney disease, highlighting key challenges for future clinical development. Nevertheless, several biological and technical challenges are yet to be addressed for broader clinical adoption of miRNA-targeted therapeutics. First, target specificity remains a major concern. A single miRNA can regulate multiple mRNAs, increasing the risk of unpredictable, potentially deleterious off-target effects. In addition, compensatory mechanisms may buffer the impact of modulating a single miRNA. Second, biological context dependence complicates interpretation and efficacy. The effects of miRNA therapies can vary by cell type, organ, disease activity, stage, and local microenvironment, leading to heterogeneous therapeutic responses. Third, efficient kidney- and cell-specific delivery remains a critical hurdle. Systemically administered oligonucleotides tend to accumulate in off-target organs such as the liver and spleen, and biological barriers within the kidney further limit access to relevant kidney cell populations, thereby constraining both efficacy and safety in vivo. To address these limitations, multiple approaches have been developed [172]. These include nanoparticle-based platforms, such as lipid nanoparticles that protect RNA cargos and improve delivery efficiency. Targeting can also be strengthened by adding ligands, such as antibodies, peptides, small molecules, or aptamers. Such ligand-guided methods may increase selectivity for kidney compartments, including the glomerulus and renal tubules. Further progress will likely involve combining optimized chemical modifications with advanced carrier engineering and kidney-targeted ligands to reduce immunogenicity and off-target effects while achieving more precise delivery to kidney cell populations.

## 12. Conclusions

Recent studies indicate shared miRNA regulators across multiple fibrotic pathways, indicating that these miRNAs are potential novel therapeutic targets for kidney interstitial fibrosis. For example, miR-21, miR-29, and miR-214 play key roles in resident fibroblast-to-myofibroblast mesenchymal transition, and miR-21, miR-27, miR-30, miR-124, and miR-214 regulate EMT.

Recent advancements in molecular diagnosis have improved our understanding of CKD with interstitial fibrosis. However, the causes of CKD remain unclear for many patients. miRNAs are promising targets for early diagnosis and therapy due to their roles in kidney function and the progression of interstitial fibrosis. Despite this potential, most preclinical findings have not translated to clinical applications. Mechanistic dissection of miRNA regulatory networks will be essential for translating experimental findings into clinical interventions.

## Figures and Tables

**Table 1:** **MiRNAs in the regulation of fibroblast-to-myofibroblast transition.**

NcRNA	Expression	Experimental Models	Targets of ncRNAs	Ref.
miR-9-5p	Down	UUO- and FA-induced mouse Mouse primary kidney fibroblasts	miR-9-5p/Pdgfrb	[56]
miR-21	Up	Kidney transplant recipients UUO-induced mouse TGF-β1-induced primary kidney fibroblasts	Not directly validated	[43]
miR-21	Up	UUO-induced mouse TGF-β1-induced NRK-49F	miR-21/Smad7 (and PDCD4/AP-1)	[44]
miR-21	Up	UUO-induced mouse Exosomes derived from TGF-β1-induced NRK-52E transfer to NRK-49F	miR-21/PTEN	[47]
miR-21a-5p	Up	Murine chronic kidney allograft dysfunction induced by allogeneic kidney transplantation NRK-49F treated with macrophage-derived small vesicles	miR-21a-5p/Notch2	[48]
miR-21-5p	Up	UUO-induced mouse TGF-β1-induced NRK-49F	miR-21-5p/PTEN and Spry1	[46]
miR-26a-5p	Down	Human kidney tissues with interstitial nephritis UUO-induced mouse TGF-β1-induced NRK-49F	Not directly validated (TGF-β and NF-κB signaling axes)	[49]
miR-29a-3p	Down	Human kidney biopsy from patients with CKD Unilateral IRI with contralateral nephrectomy and hucMSC- derived exosome injection TGF-β1-induced NRK-49F and TNF-α-induced HUVEC	miR-29a-3p/Col1a1 (fibroblasts)	[51]
miR-29b	Down	Ang II-infused mouse with rAAV9-miR-29b delivery	Not directly validated (Collagens)	[54]
miR-29c	Down	UUO-induced mouse with intrarenal pelvic injection of rAAV6-FSP1-pre-miR-29c (or inhibitor) TGF-β1-induced NRK-49F	miR-29c/TPM1	[50]
miR-29c-3p	Down	UUO-induced mouse TGF-β1-induced NRK-49F	miR-29c-3p/Fer	[53]
miR-30	Down	Human kidney tissues with fibrosis UUO- and FA-induced mouse TGF-β1-induced NRK-49F	miR-30/SOX9	[52]
miR-34a	Up	UUO-induced mouse TGF-β1-induced NRK-49F	Not directly validated (p53/miR-34a axis)	[55]
miR-122-5p	Down	UUO-induced mouse injected with exosomes isolated from TGF-β1-induced HK-2 NRK-49F treated with exosomes derived from TGF-β1-induced HK-2 cells	miR-122-5p/HIF-1α	[57]
miR-150-5p	Up	Unilateral IRI-induced mouse injected with exosomes isolated from hypoxia-treated NRK-52E NRK-49F treated with exosomes isolated from hypoxia-treated NRK-52E	miR-150-5p/SOCS1	[58]
miR-155-5p	Up	Human kidney tissues with kidney fibrosis UUO-induced rat HK-2 and NRK-49F	miR-155-5p/SOCS1 and SOCS6	[59]
miR-181	Down	Serum samples from patients with kidney fibrosis UUO-induced mouse Ang II-stimulated NRK-49F	miR-181/Egr-1	[60]
miR-184	Up	Serum samples from patients with kidney fibrosis UUO-induced mouse Ang II-stimulated NRK-49F	miR-184/HIF1AN	[61]
miR-330-3p	Up	Urine samples from patients with CKD Adenine-induced mouse with exosomes isolated from UA-treated NRK-52E NRK-49F treated with exosomes isolated from UA-treated NRK-52E	miR-330-3p/CREBBP	[62]

Ang II, angiotensin II; AP-1, activation protein-1; CKD, chronic kidney disease; Col1a1, collagen type I alpha 1 chain; CREBBP, CREB-binding protein; ECM, extracellular matrix; Egr-1, early growth response factor-1; FA, folic acid; HG, high glucose; HIF-1α, hypoxia-inducible factor 1 alpha; HIF1AN, hypoxia-inducible factor 1 subunit alpha inhibitor; HK-2, human kidney proximal tubular epithelial cell; HucMSC, human umbilical cord mesenchymal cell; HUVEC, human umbilical vein endothelial cell; IRI, ischemia–reperfusion injury; LPS, lipopolysaccharide; Notch2, neurogenic locus notch homolog protein 2; NRK-49F, normal rat kidney fibroblast cell; NRK-52E, normal rat kidney tubular epithelial cell; PDCD4, programmed cell death protein 4; Pdgfrb, platelet-derived growth factor receptor beta; PTEN, phosphatase and tensin homolog; SOX9, sex-determining region Y-box containing gene 9; rAAV, recombinant adeno-associated virus; SOCS, suppressor of cytokine signaling; Spry1, sprouty RTK signaling antagonist 1; TGF-β, transforming growth factor beta; TPM1, tropomyosin 1; UA, uric acid; UUO, unilateral ureteral obstruction.

**Table 2:** **MiRNAs in the regulation of epithelial–mesenchymal transition.**

NcRNA	Expression	Experimental Models	Targets of ncRNAs	Ref.
miR-17	Up	Serum samples from patients with DN db/db mouse TGF-β1-induced HK-2	miR-17/Smad7	[66]
miR-19	Up	Serum samples from patients with kidney fibrosis UUO-induced mouse TGF-β1-induced NRK-52E	miR-19/PTEN	[67]
miR-21	Up	Aging rat kidney PPARα-deficient mouse TGF-β1-induced NRK-52E	miR-21/PPARα	[77]
miR-21	Up	UUO-induced rat TGF-β1-induced HK-2	miR-21/PTEN	[68]
miR-21	Up	Adenine diet and FA-induced mouse LPS-stimulated NRK-52E	Not directly validated (miR-21 activates TLR7 signaling)	[78]
miR-21-5p	Up	UUO-induced mouse HK-2	Not directly validated (SPRY1/ERK/NF-κB axis)	[79]
miR-23a	Up	Kidney tissues from patients with DN HG-treated HK-2	miR-23a/SnoN	[70]
miR-27a	Up	STZ-induced rat HG-treated NRK-52E	miR-27a/Sfrp1	[74]
miR-27a-3p	Up	db/db mouse HG-treated HK-2	miR-27a-3p/Prohibitin and TMBIM6	[73]
miR-27b-3p	Down	UUO-induced mouse TGF-β1-induced HK-2	miR-27b-3p/STAT1	[75]
miR-30b	Down	HFD and STZ-induced mouse with BAT transplantation TGF-β1-induced HK-2	miR-30b/Runx1 and Snail1	[80]
miR-30b-5p	Down	Kidney tissues from patients with DN db/db mouse HG-treated HK-2	miR-30b-5p/Snail1	[81]
miR-30c	Down	db/db mouse with rAAV-miR-30c (or anti-miR-30c) HG-treated HK-2	miR-30c/Snail1	[82]
miR-32-5p	Up	STZ-induced rat HG-treated HK-2	miR-32-5p/Smad7	[83]
miR-34a-5p	Up	Kidney tissues from patients with kidney fibrosis UUO-induced mouse TGF-β1-induced HK-2	miR-34a-5p/Klotho	[84]
miR-92a-3p	Up	UUO-induced mouse TGF-β1-induced HK-2	miR-92a-3p/LIN28A	[85]
miR-92d-3p	Down	Kidney tissues from patients with DN HFD and STZ-induced mouse TGF-β1- and/or C3a-induced HK-2	Not directly validated (C3/HMGB1/TGF-β1 pathway)	[86]
miR-93	Down	Kidney tissues from patients with DN TGF-β1-induced HK-2	miR-93/Orai1	[87]
miR-101	Down	Mercury chloride (HgCl_2_)-induced rat TGF-β1-induced HK-2	miR-101/TGF-βRI	[88]
miR-124	Down	STZ-induced mouse HG-treated HK-2	miR-124/TLR4	[89]
miR-130a-3p	Up	TGF-β1-induced renal tubular epithelial cells	miR-130a-3p/SnoN	[71]
miR-130b	Down	Plasma samples and kidney tissues from patients with DN STZ-induced rat HG-treated NRK-52E	miR-130b/Snail	[90]
miR-136	Down	STZ-induced rat HG-treated NRK-52E	miR-136/SYK	[91]
miR-140-5p	Down	UUO-induced mouse TGF-β1-induced HK-2	miR-140-5p/TGF-βRI	[92]
miR-199a-3p	Up	Kidney tissues from patients with hydronephrosis UUO-induced mouse TGF-β1-induced HK-2	miR-199a-3p/Par4	[93]
miR-200a	Down	UUO-induced rat TGF-β1-induced HK-2	miR-200a/GAB1	[94]
miR-204	Down	Unilateral IRI-induced mouse Hypoxia-treated HK-2	miR-204/SP-1	[95]
miR-204	Down	HFD and STZ-induced rats with ADSC-derived exosome delivery HG-treated HK-2 and NRK-52E	miR-204/METTL7A	[96]
miR-204-5p	Down	db/db mouse with AAV9-Ksp-miR-204-5p HG-treated HK-2	miR-204-5p/Keap1	[97]
miR-214	Up	Kidney tissues from patients with DN Akita mouse and STZ-induced mouse HG-treated RPTC and BUMPT	miR-214/ULK1	[98]
miR-214	Up	Kidney tissues and urine samples from patients with CKD IRI-, UUO-, or albumin overload-induced mouse Albumin-, hypoxia-, IL-1β-, or TGF-β1-induced PTC	miR-214/mt-Nd6, mt-Nd4l	[99]
miR-302b	Down	UUO-induced mouse TGF-β1-induced HK-2	miR-302b/TGF-βRII	[100]
miR-363-3p	Down	TGF-β1-induced HK-2	miR-363-3p/TGF-β2	[101]
miR-374a-5p	Down	UUO-induced mouse with MSC-derived exosome delivery TGF-β1-induced HK-2	miR-374a-5p/MAPK6	[102]
miR-382	Up	Kidney tissues from patients with IgAN AA-induced mouse AA-treated mouse TEC	miR-382/PTEN	[69]
miR-455-3p	Down	HFD and STZ-induced rat HG- or TGF-β1-treated HK-2 or HMC	miR-455-3p/ROCK2	[103]
miR-490-3p	Down	UUO-induced mouse TGF-β1-induced NRK-52E	miR-490-3p/HMGA2	[104]
miR-494-3p	Up	HG-treated HK-2	miR-494-3p/SOCS6	[105]
miR-630	Down	STZ-induced rat HG-treated NRK-52E	miR-630/TLR4	[106]
lncRNA H19	Down	UUO-induced mouse TGF-β1-induced HK-2	lncRNA H19/miR-130a-3p/ACSL1	[76]
lncRNA XIST	Up	Kidney tissues from patients with ON UUO-induced mouse TGF-β1-induced HK-2	lncRNA XIST/miR-124-3p/ITGB1	[107]
lncRNA MALAT1	Up	Kidney tissues from patients with ON UUO-induced mouse TGF-β1-induced HK-2 and NRK-49F	lncRNA MALAT1/ miR-124-3p/ITGB1	[108]
lncRNA KCNQ1OT1	Up	UUO-induced mouse TGF-β1-induced HK-2	lncRNA KCNQ1OT1/miR-124-3p	[109]
lncRNA NORAD	Up	Tacrolimus-induced rat Tacrolimus-induced NRK-52E	lncRNA NORAD/miR-136-5p/SYK	[110]
lncRNA LUCAT1	Up	HG-treated HK-2	lncRNA LUCAT1/ miR-199a-5p/ZEB1	[111]

AA, aristolochic acid; ACSL1, long-chain acyl-CoA synthetase 1; ADSC, adipose-derived stem cells; BAT, brown adipose tissue; BUMPT, Boston University mouse proximal tubule cells; CKD, chronic kidney disease; DN, diabetic nephropathy; ERK, extracellular signal-regulated kinase; FA, folic acid; GAB1, GRB2 associated binding protein 1; HG, high glucose; HFD, high-fat diet; HK-2, human kidney proximal tubular epithelial cell; HMC, human mesangial cell; HMGA2, high mobility protein A2; IgAN, IgA nephropathy; ITGB1, integrin beta-1; IRI, ischemia–reperfusion injury; Keap1, Kelch-like ECH-associated protein 1; LIN28A, lin-28 homolog A; LPS, lipopolysaccharide; MAPK, mitogen-activated protein kinase; METTL7A, methyltransferase-like 7A; MSC, mesenchymal stem cells; NF-κB, nuclear factor kappa B; NRK-49F, normal rat kidney fibroblast cell; NRK-52E, normal rat kidney tubular epithelial cell; ON, obstructive nephropathy; Par4, prostate apoptotic response 4; PPARα, peroxisome proliferator-activated receptor α; PTC, proximal tubular cells; PTEN, phosphatase and tensin homolog; ROCK2, rho-associated coiled coil-containing protein kinase 2; RPTC, renal proximal tubular cells; Runx 1, runt-related transcription factor 1; Snail 1, snail family zinc finger 1; Sfrp1, secreted frizzled-related protein 1; SnoN, ski-related novel protein N; SOCS, suppressor of cytokine signaling; SP-1, specificity protein 1; STAT1, signal transducers and activators of transcription 1; STZ, streptozotocin; SYK, spleen tyrosine kinase; TEC, tubular epithelial cell; TGF-β, transforming growth factor beta; TGF-βRI/II, transforming growth factor beta receptor I/II; TMBIM6, transmembrane BAX inhibitor motif containing 6; TLR, toll-like receptor; ULK1, unc-51-like autophagy-activating kinase 1; UUO, unilateral ureteral obstruction; ZEB1, zinc finger E-box-binding homeobox 1.

**Table 3:** **MiRNAs in the regulation of kidney interstitial fibrosis in diabetic nephropathy.**

NcRNA	Expression	Experimental Models	Targets of ncRNAs	Ref.
miR-30a-3p	Down	db/db mouse HG-treated mouse renal tubular epithelial cells	miR-30a-3p/AIF-1/TRPC6/ calcineurin A/NFAT2	[152]
miR-543	Down	db/db mouse HG-treated HK-2	miR-543/TSPAN8	[153]
miR-23a-3p	Down	HFD- or STZ-induced mouse BSA-stimulated HK-2	miR-23a-3p/Egr1	[154]
miR-17	Up	Serum samples from patients with DN db/db mouse TGF-β1-induced HK-2	miR-17/Smad7	[66]
miR-92d-3p	Down	Kidney tissues from patients with DN HFD and STZ-induced mouse TGF-β1- and/or C3a-induced HK-2	Not directly validated (C3/HMGB1/TGF-β1 pathway)	[86]
miR-1225-3p	Up	Kidney tissues from patients with DN STZ-induced mouse HG-treated MCs	miR-1225-3p/ARHGAP5/SMURF2	[155]
miR-214-3p	Up	Kidney tissues from patients with DN STZ-induced mouse and KK-Ay/Ta mouse HG-treated MCs	miR-214-3p/DIAPH1	[156]
miR-10a/10b	Down	Kidney tissues from patients with DN STZ-induced mouse HG-treated human podocytes and HK-2	miR-10a/b/TGFBR1	[147]
miR-144-3p	Up	STZ-induced SHR TGF-β1-induced NRK-52E	miR-144-3p/NRF2	[148]
miR-365-3p	Up	STZ-induced mouse HG-treated HK-2	miR-365-3p/BDNF	[157]
Circ_000166	Up	HFD and STZ-induced mouse HG-treated HK-2	Circ_000166/miR-296/SGLT2	[158]
Circ_0054633	Up	Serum and urine samples from patients with DN db/db mouse HG-treated HRMCs	Circ_0054633/miR-136-5p/SMAD3	[149]
Circ_ITCH	Down	STZ-induced mouse HG-treated rat MCs	Circ_ITCH/miR-33a-5p/SIRT6	[159]
LncRNA H19	Up	STZ-induced CD-1 mouse TGF-β2-induced HMVECs	LncRNA H19/miR-29a/ TGF-β/Smad signaling	[160]
LncRNA SNHG14	Up	STZ-induced mouse HG-treated human MCs	LncRNA SNHG14/miR-30e-5p/SOX4	[150]
LncRNA TUG1	Down	STZ-induced mouse with AAV-TUG1 delivery HG-treated HK-2	LncRNA TUG1/miR-145-5p/DUSP6	[161]
LncRNA GAS5	Down	db/db mouse	LncRNA GAS5/miR-542-3p/ERBB4	[162]

AIF-1, allograft inflammatory factor 1; ARHGAP5, Rho GTPase-activating protein 5; BDNF, brain-derived neurotrophic factor; BSA, bovine serum albumin; DIAPH1, diaphanous related formin 1; DN, diabetic nephropathy; DUSP6, dual-specificity phosphatase 6; Egr1, early growth response 1; ERBB4, erb-b2 receptor tyrosine kinase 4; HFD, high-fat diet; HG, high glucose; HK-2, human kidney proximal tubular epithelial cell; HMVECs, human microvascular endothelial cells; HRMCs, human renal mesangial cells; MCs, mesangial cells; NFAT2, nuclear factor of activated T-cells 2; NRF2, nuclear factor erythroid 2–related factor 2; SHR, spontaneously hypertensive rat; SGLT2, sodium–glucose cotransporter 2; SIRT6, sirtuin 6; SMAD3, SMAD family member 3; SMURF2, SMAD specific E3 ubiquitin protein ligase 2; SOX4, SRY-box transcription factor 4; STZ, streptozotocin; TGF-β, transforming growth factor beta; TSPAN8, tetraspanin 8; TRPC6, transient receptor potential cation channel subfamily C member 6.

## Data Availability

No new data were created or analyzed in this study. Data sharing is not applicable to this article.

## Abbreviations

The following abbreviations are used in this manuscript:

AA	Aristolochic acid
ACSL1	Acyl-CoA synthetase long-chain family member 1
AKI	Acute kidney injury
α-SMA	Alpha-smooth muscle actin
Ang II	Angiotensin II
AP-1	Activator protein 1
BAT	Brown adipose tissue
BAX	BCL2-associated X protein
BUMPT	Boston University mouse proximal tubule cells
CBM	Coalbed methane
CD31	Cluster of differentiation 31
CD68	Cluster of differentiation 68
CKD	Chronic kidney disease
COL1A1	Collagen type I alpha 1 chain
CREBBP	CREB-binding protein
CTGF	Connective tissue growth factor
circRNA	Circular RNA
DN	Diabetic nephropathy
ECM	Extracellular matrix
EMT	Epithelial–mesenchymal transition
EndMT	Endothelial–mesenchymal transition
ER	Endoplasmic reticulum
ESKD	End-stage kidney disease
EV	Extracellular vesicle
FA	Folic acid
FGF2	Fibroblast growth factor 2
FUT8	α1,6-fucosyltransferase
GAB1	GRB2-associated binding protein 1
GSAP	γ-secretase–activating protein
HG	High glucose
HIF-1α	Hypoxia-inducible factor 1 alpha
HK-2	Human kidney proximal tubular epithelial cell line
HUVEC	Human umbilical vein endothelial cell
IF/TA	Interstitial fibrosis/tubular atrophy
IL	Interleukin
iNOS	Inducible nitric oxide synthase
IRI	Ischemia–reperfusion injury
JAK	Janus kinase
LNA	Locked nucleic acid
lncRNA	Long non-coding RNA
MAPK	Mitogen-activated protein kinase
METTL7A	Methyltransferase-like 7A
miRNA/miR	MicroRNA
miRISC	MicroRNA-induced silencing complex
MMT	Macrophage–mesenchymal transition
MMP	Matrix metalloproteinase
NF-κB	Nuclear factor kappa B
NRK-49F	Normal rat kidney fibroblast cell line
NRK-52E	Normal rat kidney tubular epithelial cell line
PAS	Periodic acid–Schiff
PDCD4	Programmed cell death protein 4
PDGFR-β	Platelet-derived growth factor receptor beta
PI3K	Phosphoinositide 3-kinase
PPARα	Peroxisome proliferator-activated receptor alpha
PTEN	Phosphatase and tensin homolog
PTFS	Pure total flavonoids from *Smilax glabra*
rAAV	Recombinant adeno-associated virus
RBP	RNA-binding protein
RFA	Radiofrequency ablation
RISC	RNA-induced silencing complex
RUNX1	Runt-related transcription factor 1
SARA	Smad anchor for receptor activation
Sfrp1	Secreted frizzled-related protein 1
SIRP-α	Signal regulatory protein alpha
SMAD	Mothers against decapentaplegic homolog
SnoN	Ski-related novel protein N
SOCS1	Suppressor of cytokine signaling 1
SPRY1	Sprouty homolog 1
STAT	Signal transducer and activator of transcription
STZ	Streptozotocin
TGF-β	Transforming growth factor beta
TGF-βRI/II	Transforming growth factor beta receptor I/II
TLR4	Toll-like receptor 4
TNF	Tumor necrosis factor
TPM1	Tropomyosin 1
TUG1	Taurine upregulated gene 1
UUO	Unilateral ureteral obstruction
VE-cadherin	Vascular endothelial cadherin
VEGF	Vascular endothelial growth factor
vWF	von Willebrand factor
